# Gailbraith A. Irvine, M.D.

**Published:** 1867-05

**Authors:** T. F. Parker

**Affiliations:** Warren


DIED—At Warren, Pa., February 11th, 1867, GAILBRAITH A. IRVINE, M. D., aged 57 years.

Dr. G. A. Irvine was born in Sunbury, Pa., July 1, 1811, and was from one of the most ancient and respectable families in the State; Gen. Irvine of the Revolution, being his grandfather. He early chose that profession which was the first command of our Savior to his disciples, "heal the sick," graduated at the University of Pennsylvania, and was appointed Physician and Surgeon to the Philadelphia Alms House. His health failing in that position, he removed to this county in 1838, which became his residence until his death.

Dr. Irvine was one of the Curators of the Buffalo Medical College, and by his devotion to his profession, of which he was an honor and ornament, won for himself an extensive acquaintance throughout the State. He was generous to the poor, a firm believer in the doctrines of the Bible, kind and affectionate as a father and husband, and has left a lovely and amiable family who mourn his loss, and refuse to be comforted, because "he is not." We hope it may be said to him, "I was sick and ye visited me."

T. F. PARKER, M. D.

Warren, May 14, 1867.

